# Potential biomarkers and immune characteristics of small bowel adenocarcinoma

**DOI:** 10.1038/s41598-022-20599-5

**Published:** 2022-09-28

**Authors:** Jinggao Feng, Xiayu Tang, Liusong Song, Zhipeng Zhou, Yuan Jiang, Yao Huang

**Affiliations:** Department of Gastrointestinal and Anorectal Surgery, The Central Hospital of Yongzhou, No. 151, Xiaoshui West Road, Lingling District, Yongzhou, 425100 Hunan China

**Keywords:** Cancer, Genetics, Biomarkers, Gastroenterology, Oncology

## Abstract

Small bowel adenocarcinoma (SBA) is a gastrointestinal malignancy with low incidence but poor prognosis, and its pathogenesis is still unclear. This study aimed to explore potential disease-causing biomarkers of SBA. The gene expression datasets of SBA and normal samples were downloaded from the Gene Expression Omnibus database. First, differential gene expression analysis and weighted gene coexpression network analysis (WGCNA) were performed. Common genes (CGs) were obtained by intersection of differentially expressed genes (DEGs) and optimal modal genes of WGCNA. Subsequently, a protein‒protein interaction network was established to screen hub genes, and target genes were obtained by Lasso regression analysis of hub genes. An SBA risk prediction model was established based on target genes. The prediction accuracy of the model was evaluated by the area under the receiver operating characteristic curve (AUC). The levels of immune cell infiltration and activation of immune pathways were compared between SBA and normal samples using the "ggpubr" and "reshape2" packages. A total of 1058 DEGs were identified. WGCNA showed that the signature gene in the brown module was significantly associated with SBA (p = 7E−17), and 469 CGs were obtained. Four target genes (APOA4, APOB, COL1A2, FN1) were identified and showed excellent prediction of SBA risk (AUC = 0.965). In addition, active dendritic cells and macrophages showed higher infiltration levels in SBA. Meanwhile, the APC_co_stimulation pathway and parainflammation pathway were strongly active in SBA. Four target genes (APOA4, APOB, COL1A2, FN1) may be involved in the pathogenesis of small bowel adenocarcinoma.

## Introduction

As a rare tumor, small bowel adenocarcinoma (SBA) accounts for only 3–5% of digestive malignant tumors^1^. In 2021, there were 11,390 new cases of SBA in the United States and 2100 deaths from the disease^2^. Because of the nonspecificity of SBA symptoms, the diagnosis is often due to intestinal perforation, ileus, and uncontrolled gastrointestinal bleeding. Therefore, approximately one-third of patients are diagnosed with distant metastasis^3^. Meanwhile, the prognosis of SBA is poor, especially for patients in an advanced stage^4,5^.

Currently, the understanding of the pathogenesis of SBA is still limited. The traditional concept is that the pathogenesis of SBA is similar to that of colon cancer but lacks exact evidence. Several key molecular drivers in the pathogenesis of SBA have been identified by genomic profiling studies, including TP53, SMAD4, KRAS and E-cadherin^6,7^. Fortunately, many studies have reported that the incidence of high microsatellite instability (MSI-H) or dMMR and high tumor mutational burden (TMB) in SBAs is higher than that in gastric cancer and colorectal cancer, suggesting that immunotherapy may be a new therapeutic breakthrough^8–10^. However, the state of immune cell infiltration and immune pathway activation in the immune microenvironment in SBA is still unclear. Hereditary cancer syndromes such as Lynch syndrome, familial adenomatous polyposis and Peutz‒Jeghers syndrome are considered to be risk factors for SBA^11,12^. In addition, patients with Crohn's disease and celiac disease are more likely to suffer from SBA^13,14^.

To date, few studies on potential biomarkers of SBA have been recorded. In this study, comprehensive bioinformatics methods were utilized to explore the pathogenesis of SBA and screen biomarkers that may have therapeutic value. Meanwhile, the tumor immune microenvironment of SBA was discussed.

## Methods

### Patient and public involvement

The dataset (GSE61465) downloaded for this study contains 20 normal samples and 25 small bowel adenocarcinoma samples. The GEO database belongs to the public databases. The patients involved in the database obtained ethical approval. Users can download relevant data for free for research and publish relevant articles. Our study is based on open source data, so there are no ethical issues or other conflicts of interest.

The statistical analysis of this study was completed by R version 4.1.0 (http://www.r-project.org). P < 0.05 on both sides was considered statistically significant. The Gene Expression Omnibus (GEO) database (https://www.ncbi.nlm.nih.gov/geo/) was used to download the gene expression dataset for SBA pathological tissues and normal small bowel mucosa samples^15^. Data preprocessing was performed on the dataset using the "limma" and "impute" packages, including conversion of probe names to gene names, missing value filling and data normalization^16^. Deleted data with unrecognized gene names. Genes satisfying FDR < 0.01 and |log2 -fold change (FC)|> 2 were selected through the "limma" package, and these genes were designated differentially expressed genes (DEGs)^17^.

Weighted gene coexpression network analysis (WGCNA) was used to explore the interaction between DEGs. The gene coexpression network was constructed by the "WGCNA" package^18^. First, genes with more than 25% variation between samples were introduced into WGCNA. Second, the soft threshold was calculated by the pickSoftThreshold function, and RsquaredCut was set to 0.9^19^. The best soft threshold was chosen, and the adjacency matrix was calculated. Then, the adjacency matrix was converted into a topological overlap matrix (TOM), and the degree of dissimilarity between genes was calculated. Third, gene modules were divided using the dynamic shear tree, the minimum gene module size was set to 50, and then the modules with a dissimilarity coefficient less than 0.2 were merged^20^. Fourth, we selected the module associated with clinical traits, calculated the relationship between genes and traits and modules, and then visualized the characteristic gene network. The intersection genes between DEGs and genes in important modules were defined as common genes (CGs).

CGs were uploaded to the DAVID database (https://david.ncifcrf.gov/tools.jsp) and KOBAS database (http://kobas.cbi.pku.edu.cn/genelist/), and the significantly enriched Gene Ontology (GO) analysis and Kyoto Encyclopedia of Genes and Genomes (KEGG) analysis results were exported, and the results were visualized^21,22^. In addition, CGs were imported into the STRING database (https://www.string-db.org/)^23^, protein‒protein interaction (PPI) networks were built, the minimum required interaction score was set to 0.9, the results were exported, and Cytoscape Version 3.8.2 was applied to visualize the PPI network^24^. The CytoHubba plug-in in Cytoscape was used to calculate the degree of each CG node and screen out the top 10 hub genes^25^.

The "glmnet" package was used for logistic LASSO regression analysis of hub genes, and hub genes with a strong correlation to the risk of SBA were obtained and defined as target genes^26^. The "rms" package was used to draw the nomogram of the model for predicting SBA risk based on the above hub genes^27^. A receiver operating characteristic (ROC) curve was obtained by the "ROCR" package^28^. ROC curves were used to evaluate the prediction accuracy of the model.

The levels of immune cell infiltration and activation of the immune pathway in the SBA and control groups were analyzed by using the "ggpubr" and "reshape2" packages, respectively. In addition, the "corrplot" package was used to explore the relationships between immune cells and between immune pathways.

The miRTarBase, StarBase and TargetScan databases were used to predict the microRNAs (miRNAs) of CGs^29–31^. The miRNAs obtained from the three databases were intersected to obtain the target miRNAs. On the other hand, CGs were imported into the Enrichr database (https://maayanlab.cloud/Enrichr/)^32^, and transcription factors (TFs) targeting CGs with p < 0.01 were screened out to obtain the TF-mRNA regulatory network. The above regulatory networks were visualized by Cytoscape.

### Ethical approval and consent to participate

The Ethics Committee of The Central Hospital of Yongzhou reviewed the study, and ethics approval was not necessary.

## Results

### Identification of DEGs

After filtering, one dataset (GSE61465) was downloaded from the GEO database. By analyzing the expression levels of genes in the above dataset, 1058 DEGs were obtained, of which 383 were upregulated and 675 were downregulated.

### Identification of gene coexpression networks and modules

**Figure 1:**
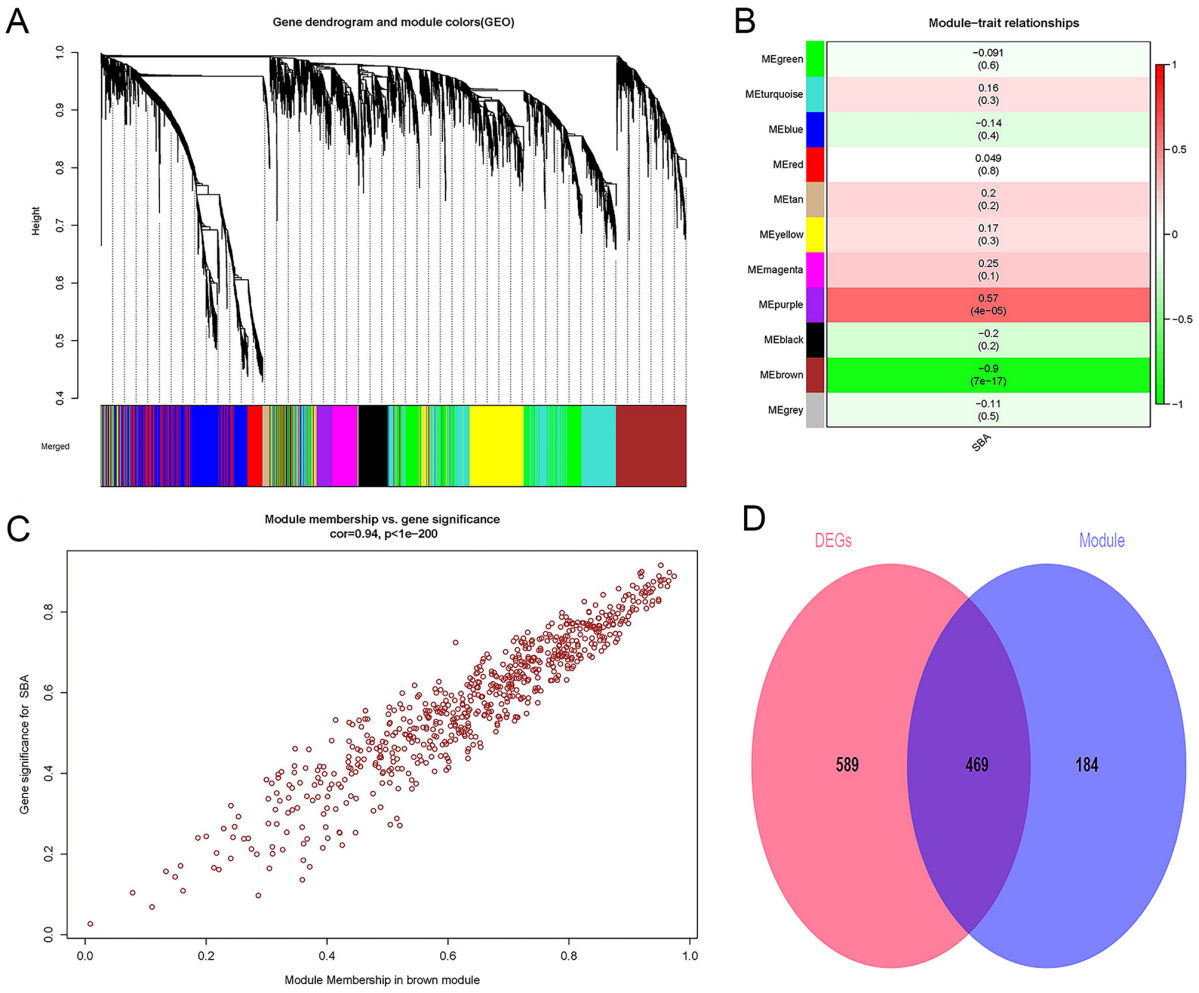
Weighted gene co-expression network analysis and Venn diagram. (**A**) Gene co-expression modules, represented by different colors under the gene tree. (**B**) Heatmap of the association between modules and SBA. The brown module was significantly correlated with SBA. The numbers inside and outside of the brackets represent p-values and correlation coefficients, respectively. (**C**) Correlation plot between MM (X-axis) and (GS) (Y-axis) of genes contained in the blue module. (**D**) Venn diagram showing overlapping genes between the DEGs and the genes in the brown module. *SBA* small bowel adenocarcinoma, *GS* gene significance, *MM* module membership. Color images are available online; DEGs, differentially expressed genes.

First, 25% (5182) of the genes with the largest variance were extracted for subsequent analysis. Second, we defined the threshold to 50 for cluster analysis. Third, R^2^ was set to 0.9, and the best soft threshold was 6. Fourth, genes with a dissimilarity coefficient less than 0.2 were combined to obtain 11 modules, and the genes in each module had similar coexpression traits (Fig. 1A). Eleven modules were randomly distinguished by color. The characteristic gene (ME) in the brown module (r = − 0.9; p = 7E−17) showed the highest positive correlation and the most significant correlation with SBA (Fig. 1B). Meanwhile, there was a significant positive relationship between the module members of the genes (MMs, the correlation between specific genes and the characteristic genes of the module) in the brown module and the gene significance (GSs, the correlation between specific genes and clinical variables). A significant correlation (cor = 0.94, P < 1E−200) was observed, as shown in Fig. 1C. Finally, the DEGs and the genes in the brown module were intersected to obtain CGs (Fig. 1D). Furthermore, the brown module was determined to be the key module of SBA, and 653 genes contained in the module were used for the next analysis. The number of genes in each analysis phase is shown in Table 1.

**Table 1:** The number of genes in each phase of the analysis. DEGs, differentially expressed genes; WGCNA, weighted gene coexpression network analysis.

Characteristics	Gene number
Original dataset	20,727
DEGs	1058
Input to WGCNA	20,727
Brown gene	653

### Enrichment analyses of the common genes and hub genes

**Figure 2:**
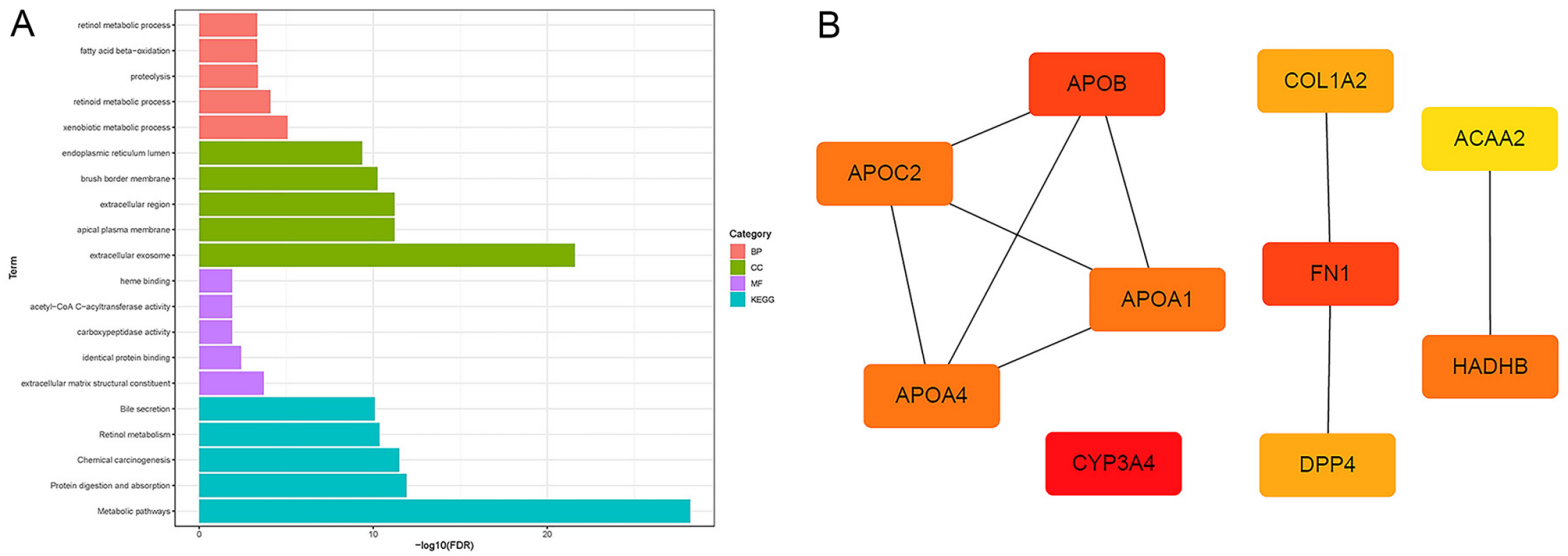
GO analysis, KEGG pathway analysis and hub genes. (**A**) The pink bars represent biological processes, the green bars represent cellular components, the purple lines represent molecular functions, and the blue lines represent KEGG pathways. (**B**) Hub genes. *BP* biological process, *CC* cellular component, *MF* molecular function, *GO* Gene Ontology, *KEGG* Kyoto Encyclopedia of Genes and Genomes.

In biological processes (BP), CGs were mainly enriched in "xenobiotic metabolic process", "retinoid metabolic process", "proteolysis", "fatty acid beta-oxidation" and "retinol metabolic process". CGs are mainly involved in the cell component (CC) ontology, including "extracellular exosome", "apical plasma membrane", "extracellular region", "brush border membrane" and "endoplasmic reticulum lumen". In molecular function (MF), CGs were mostly enriched in "extracellular matrix structural constituent" and "identical protein binding". In addition, the KEGG enrichment pathways included "Metabolic pathways", "Protein digestion and absorption", "Chemical carcinogenesis", "Retinol metabolism" and "Bile secretion". All enrichment pathways are shown in Fig. 2A. Ten hub genes (APOB, APOC2, APOA4, APOA1, CYP3A4, COL1A2, FN1, DPP4, ACAA2, HADHB) are shown in Fig. 2B.

### SBA risk prediction model

**Figure 3:**
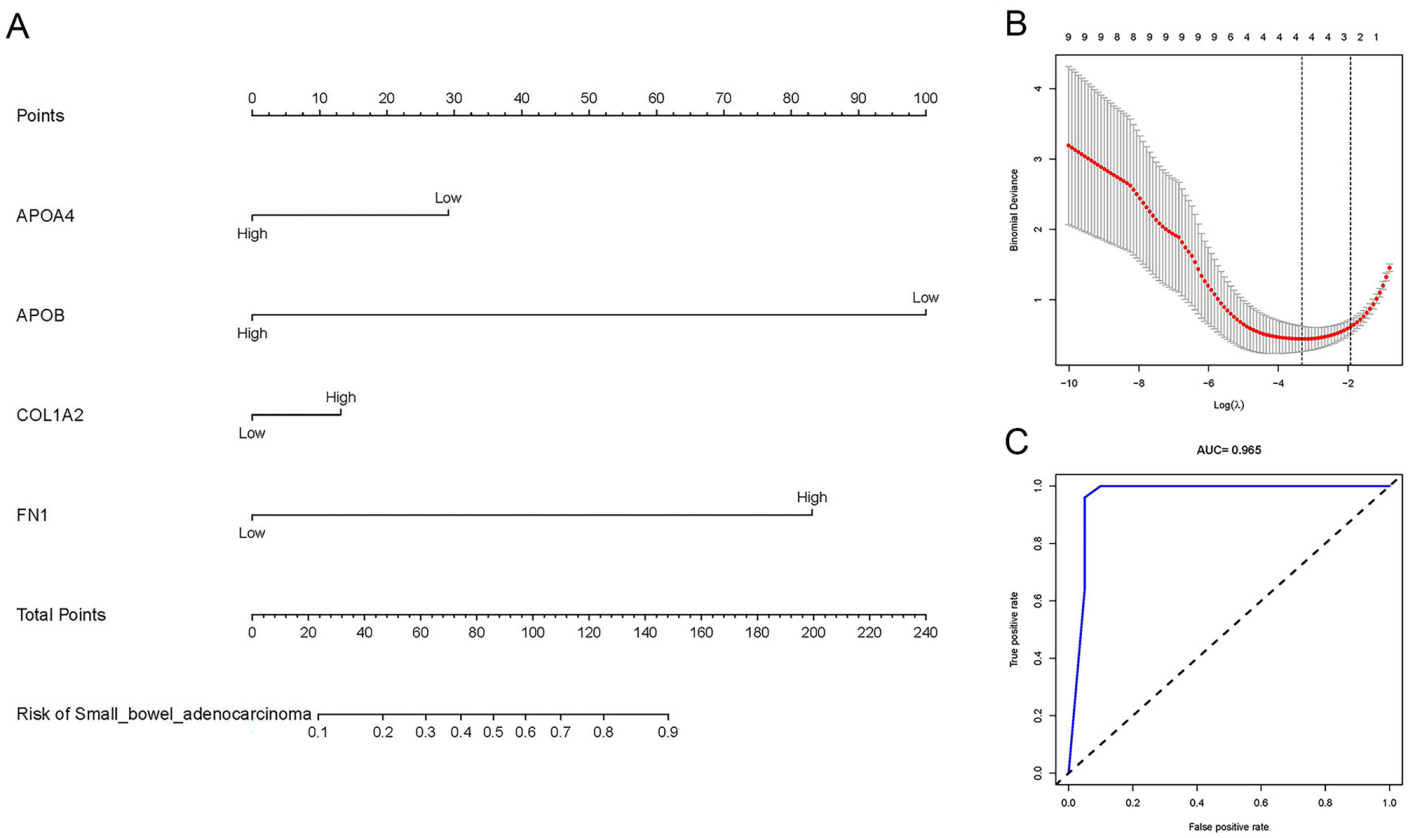
SBA risk prediction model based on 4 target genes. (**A**) SBA risk prediction model based on four target genes. (**B**) Optimal parameter (lambda) selection in the LASSO model used fivefold cross-validation via minimum criteria. The partial likelihood deviance (binomial deviance) curve was plotted versus log(lambda). Dotted vertical lines were drawn at the optimal values by using the minimum criteria and the 1 SE of the minimum criteria (the 1-SE criteria). (**C**) ROC Curves. *SBA* small bowel adenocarcinoma, *LASSO* least absolute shrinkage and selection operator, *SE* standard error, *ROC* receiver operating characteristic.

Four target genes (APOA4, APOB, COL1A2, FN1) related to the risk of SBA were obtained through LASSO regression analysis, as shown in Fig. 3B. The SBA risk prediction model based on the above four hub genes is shown in Fig. 3A. The ROC curve is shown in Fig. 3C. The area under the curve (AUC) was 0.965, indicating that the model has excellent prediction accuracy.

### Immune infiltration analysis

**Figure 4:**
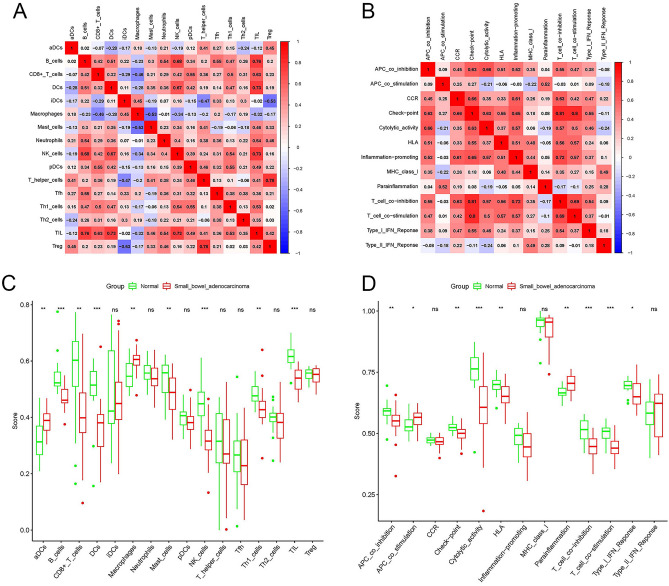
Characteristics of the immune microenvironment in SBA. (**A**) Correlation between immune cells in SBA. Red represents a positive correlation, blue represents a negative correlation. A deeper color indicates a stronger correlation. (**B**) Correlation between immune pathways in SBA. Red represents a positive correlation, blue represents a negative correlation. A deeper color indicates a stronger correlation. (**C**) Comparison of the enrichment scores of 16 types of immune cells between the SBA (red box) and normal group (green box). (**D**) Comparison of the enrichment scores of 13 immune-related pathways between the SBA (red box) and normal group (blue box). *SBA* small bowel adenocarcinoma, P values: *ns* not significant; *P < 0.05; **P < 0.01; ***P < 0.001.

Figure 4A,B show the relationship between immune cells and between immune pathways, respectively. In Fig. 4A, the infiltration of tumor infiltrating lymphocytes (TILs) was positively correlated with the infiltration of B cells, dendritic cells (DCs), and natural killer (NK) cells. In addition, T regulatory (Treg) cell infiltration was positively correlated with T helper cell infiltration. The check-point pathway was positively correlated with the T_cell_co-inhibition pathway and T_cell_co-stimulation pathway, as shown in Fig. 4B. Figure 4C,D show the level of immune cell infiltration and activation of immune pathways in the SBA and control groups, respectively. Compared with the control group, the infiltration levels of B cells, CD8+-T cells, DCs, mast cells, NK cells, type 1 T helper (Th1) cells and TILs were lower in SBA. However, active dendritic cells (aDCs) and macrophages showed higher infiltration levels in SBA. Meanwhile, in the control group, the activation of the APC_co_inhibition pathway, Check-point pathway, Cytolytic_activity pathway, Human leukocyte antigen (HLA) pathway, T_cell_co-inhibition pathway, T_cell_co-stimulation pathway and Type_I_IFN_Response pathway was stronger than that of SBA. Activation of the APC_co_stimulation pathway and the parainflammation pathway of SBA was stronger than that of the control group.

### Target miRNAs and TF‐mRNA regulatory network analysis

**Figure 5:**
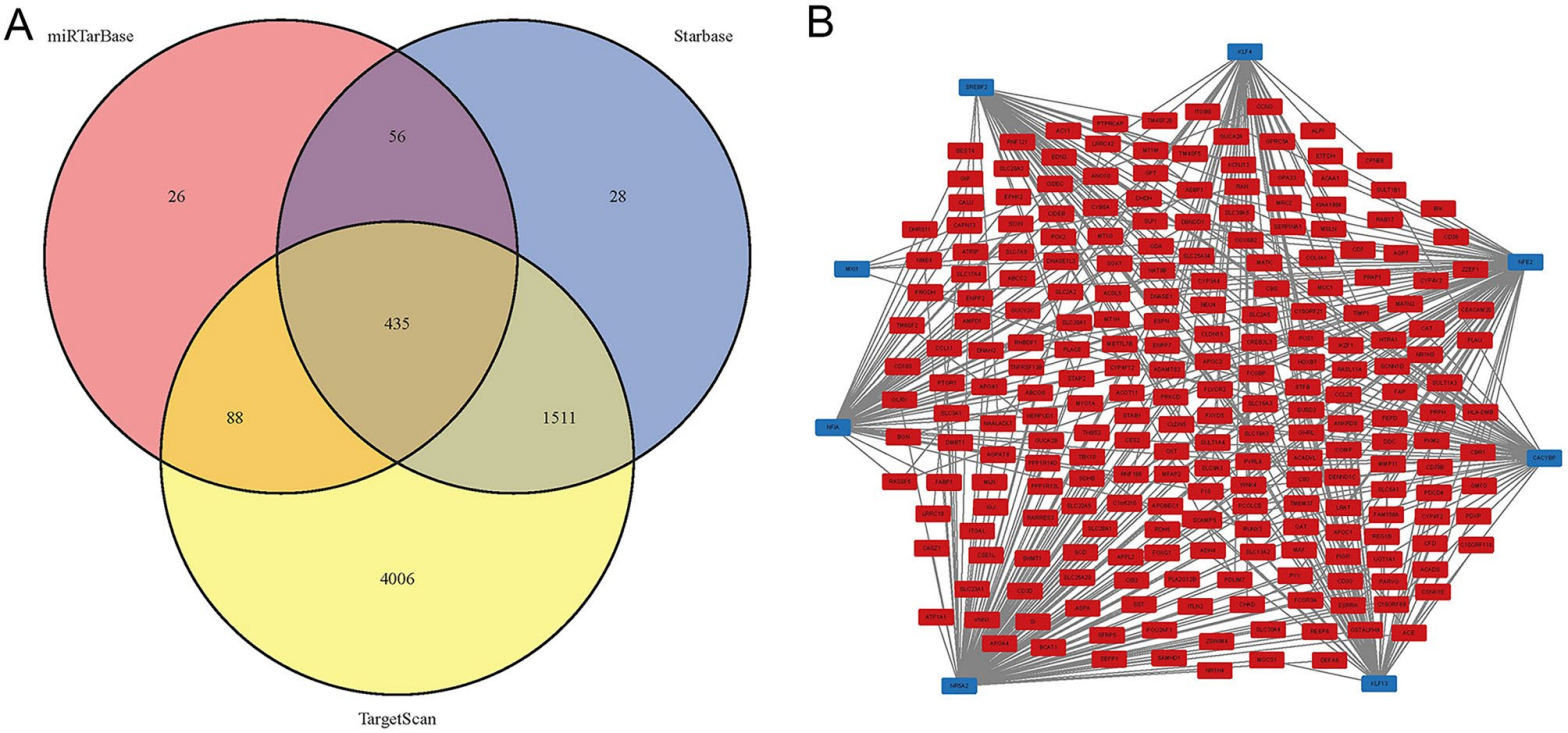
The target miRNA and TF‐mRNA regulatory network. (**A**) The Venn diagram of target miRNAs. (**B**) Red represents genes, blue represents TFs. *mRNAs* messenger RNAs, *miRNAs* microRNAs, *TFs* transcription factors.

Figure 5A shows the Venn diagram of predicted miRNAs. This study predicted 435 target miRNAs that may be involved in SBA occurrence (Supplementary Material S1). Meanwhile, 8 TFs (NFIA, KLF13, MXI1, CACYBP, NFE2, SREBF2, KLF4, NR5A2) that may be involved in the pathogenesis of SBA are shown in Fig. 5B.

## Discussion

SBA is a malignant tumor with low incidence but poor prognosis. In this study, the messenger RNAs (mRNAs), miRNAs and TFs that may be associated with its occurrence were predicted using a comprehensive bioinformatics analysis. Meanwhile, the SBA risk prediction model based on hub genes with good prediction accuracy was established. Finally, analysis of the tumor immune microenvironment suggested that the invasion level of most immune cells in SBA was low, and the activation intensity of the immune pathway was weak, which might be the cause of tumor progression.

Hub genes have attracted extensive attention as potential drug targets. In this study, four hub genes (APOA4, APOB, COL1A2, FN1) were found to be significantly related to the pathogenesis of SBA. Apolipoprotein A4 (APOA4) encodes apolipoprotein A-IV, which is hydrolyzed and glycosylated to produce acidic glycoproteins mainly found in chylomicrons (CMs), very low-density lipoprotein (VLDL), and high-density lipoprotein (HDL)^33–35^. It plays an important role in lipid transport and metabolism, especially in cholesterol reversal^36^. Recent studies showed that APOA4 was significantly overexpressed in Helicobacter pylori-infected atrophic gastritis and intestinal metaplasia tissues, as well as gastric cancer tissues^37^. Furthermore, APOA4 is considered a diagnostic marker for colorectal cancer^38,39^. As a metabolic gene, apolipoprotein B (APOB) is the most important apolipoprotein on chylomicrons and low-density lipoprotein^40,41^. APOB has been confirmed to be associated with the pathogenesis of a variety of gastrointestinal malignancies, including liver cancer, gallbladder cancer, esophageal cancer and pancreatic duct adenocarcinoma^42–45^. Abdominal obesity has been identified as a risk factor for SBA^46^. In this study, APOA4 and APOB were significantly downregulated in SBA, suggesting that lipid metabolism disorders may play an important role in the occurrence of SBA. In addition, functional enrichment analysis showed that CGs were enriched in multiple metabolically related pathways. Unfortunately, no studies have been found on APOA4 or APOB and SBA. Therefore, further research is necessary to clarify the role of lipid metabolism in SBA.

Collagen, Type I, Alpha 2 (COL1A2) is distributed in collagen and cytoplasm and is involved in bone development and the signal transduction pathway of transmembrane receptor protein tyrosine kinase^47,48^. COL1A2 was confirmed to be significantly overexpressed in gastric cancer tissues^49–51^. Similarly, COL1A2 was believed to be significantly overexpressed in colorectal cancer tissues and blood samples, but the specific mechanism remains unclear^52,53^. Increasing evidence shows that COL1A2 is considered to be a diagnostic and prognostic biomarker due to its significant upregulation in many cancers^54–56^. In contrast, COL1A2 was significantly downregulated in bladder cancer, malignant melanoma and head and neck cancer^57–59^. In this study, COL1A2 was significantly overexpressed in SBA. Fibronectin 1 (FN1) is a glycoprotein distributed in the extracellular matrix and plays an important role in carcinogenesis and metastasis^60–62^. FN1 expression was upregulated by the transcription factor CP2, which is involved in the metastasis of hepatocellular carcinoma^63^. FN1 promotes ovarian cancer metastasis by activating the PI3K/Akt pathway^64^. High expression of FN1 has been shown to be carcinogenic in esophageal cancer^65^. In addition, FN1, as an oncogene, is involved in aggressive and poor prognosis of colon cancer^66^. As expected, high expression of FN1 was significantly associated with poorer prognosis of gastric cancer^67,68^. This study suggests that the highly expressed FN1 plays an important role in the carcinogenesis of SBA.

As innate immune cells, macrophages play an important role in the tumor microenvironment. High macrophage infiltration has been associated with tumor progression or poor prognosis in a variety of solid tumors, including neck squamous cell carcinoma, gliomas, breast cancer, bladder cancer, prostate cancer, and melanoma^69–74^. Conversely, high macrophage infiltration was associated with a better prognosis of gastrointestinal malignancies, such as gastric cancer and colorectal cancer^75^. This study suggested that the level of macrophage infiltration in SBA was low, which may be one of the reasons for the poor prognosis of SBA. In addition, the low infiltration of most immune cells and the weak activation of immune pathways may be important factors for the occurrence and progression of SBA.

Although the present study is novel and rigorous, there are still some shortcomings. First, the data used in this study were obtained from a common public database, and because of the low prevalence of SBA, we were unable to collect sufficient samples for experiments to validate the results of this study. Second, due to the lack of prognostic information on SBA, our study could not be further investigated in the context of prognosis. In view of this, we hope that more studies in the future will further validate our results and investigate them in depth.

In conclusion, four mRNAs (APOA4, APOB, COL1A2, FN1) were predicted to be associated with the occurrence of SBA, and an excellent SBA risk prediction model was established based on these genes. Meanwhile, in SBA, it is speculated that the infiltration level of immune cells was low and the activation state of immune pathways was weak. Finally, TFs and miRNAs that may be involved in the pathogenesis of SBA were predicted.

## Discussion

This study predicted that four target mRNAs (APOA4, APOB, COL1A2, FN1) might be involved in the occurrence and progression of SBA. In addition, low infiltration of immune cells and weak activation of immune pathways may be immunological characteristics of SBA.

## Supplementary Information

Supplementary Information.

## Data Availability

The datasets (GSE61465) generated and analyzed during the current study are available in the Gene Expression Omnibus (GEO) repository (https://www.ncbi.nlm.nih.gov/geo/query/acc.cgi?acc=GSE61465).
