# Supplementary material for: Potential biomarkers and immune characteristics of small bowel adenocarcinoma
Source: Sci Rep. 2022 Sep 28;12:16204. doi: 10.1038/s41598-022-20599-5 (PMC9519963; doi:10.1038/s41598-022-20599-5)
Supplement: Supplementary file 1 — Supplementary Information. [file 41598_2022_20599_MOESM1_ESM.docx]

**Target miRNAs**

hsa-miR-15b-5p

hsa-miR-183-5p

hsa-miR-9-5p

hsa-miR-128-3p

hsa-miR-433-3p

hsa-miR-761

hsa-miR-105-5p

hsa-miR-525-5p

hsa-miR-33a-5p

hsa-miR-103a-3p

hsa-miR-1286

hsa-miR-3140-3p

hsa-miR-153-3p

hsa-miR-449c-5p

hsa-miR-3909

hsa-miR-576-5p

hsa-miR-5586-5p

hsa-miR-588

hsa-miR-5579-3p

hsa-miR-107

hsa-miR-579-3p

hsa-miR-497-5p

hsa-miR-409-3p

hsa-miR-338-3p

hsa-miR-34c-5p

hsa-miR-450b-5p

hsa-miR-513b-5p

hsa-miR-34a-5p

hsa-miR-214-3p

hsa-miR-34b-5p

hsa-miR-1252-5p

hsa-miR-15a-5p

hsa-miR-485-5p

hsa-miR-3619-5p

hsa-miR-449a

hsa-miR-195-5p

hsa-miR-424-5p

hsa-miR-16-5p

hsa-miR-543

hsa-miR-449b-5p

hsa-miR-1297

hsa-miR-340-5p

hsa-let-7d-5p

hsa-let-7e-5p

hsa-miR-5000-3p

hsa-miR-362-3p

hsa-miR-423-5p

hsa-miR-488-3p

hsa-miR-26b-5p

hsa-miR-3529-5p

hsa-miR-140-5p

hsa-let-7c-5p

hsa-let-7f-5p

hsa-miR-379-5p

hsa-miR-513c-5p

hsa-miR-5194

hsa-miR-542-3p

hsa-let-7i-5p

hsa-let-7g-5p

hsa-miR-506-5p

hsa-miR-98-5p

hsa-miR-329-3p

hsa-miR-944

hsa-let-7a-5p

hsa-miR-490-3p

hsa-let-7b-5p

hsa-miR-362-5p

hsa-miR-26a-5p

hsa-miR-629-5p

hsa-miR-552-3p

hsa-miR-3179

hsa-miR-146a-5p

hsa-miR-372-3p

hsa-miR-19b-3p

hsa-miR-873-5p

hsa-miR-520c-3p

hsa-miR-526b-5p

hsa-miR-493-3p

hsa-miR-323a-3p

hsa-miR-302c-3p

hsa-miR-302d-3p

hsa-miR-539-3p

hsa-miR-556-3p

hsa-miR-142-3p

hsa-miR-577

hsa-miR-147a

hsa-miR-146b-5p

hsa-miR-512-3p

hsa-miR-589-5p

hsa-miR-221-3p

hsa-miR-520g-3p

hsa-miR-106b-5p

hsa-miR-129-5p

hsa-miR-196b-5p

hsa-miR-302a-3p

hsa-miR-23b-3p

hsa-miR-200b-3p

hsa-miR-519d-3p

hsa-miR-1294

hsa-miR-3605-5p

hsa-miR-20a-5p

hsa-miR-200c-3p

hsa-miR-135a-5p

hsa-miR-520f-3p

hsa-miR-1224-5p

hsa-miR-302e

hsa-miR-373-3p

hsa-miR-302b-3p

hsa-miR-487a-3p

hsa-miR-599

hsa-miR-429

hsa-miR-218-5p

hsa-miR-205-5p

hsa-miR-192-5p

hsa-miR-20b-5p

hsa-miR-106a-5p

hsa-miR-485-3p

hsa-miR-1185-5p

hsa-miR-212-3p

hsa-miR-17-5p

hsa-miR-374b-5p

hsa-miR-222-3p

hsa-miR-215-5p

hsa-miR-23a-3p

hsa-miR-154-3p

hsa-miR-19a-3p

hsa-miR-219a-2-3p

hsa-miR-134-5p

hsa-miR-758-3p

hsa-miR-520d-3p

hsa-miR-3163

hsa-miR-519b-3p

hsa-miR-132-3p

hsa-miR-31-5p

hsa-miR-520a-3p

hsa-miR-196a-5p

hsa-miR-93-5p

hsa-miR-204-5p

hsa-miR-211-5p

hsa-miR-665

hsa-miR-301b-3p

hsa-miR-150-5p

hsa-miR-130a-3p

hsa-miR-301a-3p

hsa-miR-199a-5p

hsa-miR-346

hsa-miR-130b-3p

hsa-miR-2116-3p

hsa-miR-199b-5p

hsa-miR-454-3p

hsa-miR-122-5p

hsa-miR-532-3p

hsa-miR-2355-5p

hsa-miR-365a-3p

hsa-miR-1-3p

hsa-miR-135b-5p

hsa-miR-431-5p

hsa-miR-513a-5p

hsa-miR-18a-5p

hsa-miR-27a-3p

hsa-miR-369-3p

hsa-miR-384

hsa-miR-18b-5p

hsa-miR-151a-3p

hsa-miR-30e-5p

hsa-miR-325

hsa-miR-30a-5p

hsa-miR-30b-5p

hsa-miR-27b-3p

hsa-miR-10a-5p

hsa-miR-613

hsa-miR-670-3p

hsa-miR-23c

hsa-miR-10b-5p

hsa-miR-370-3p

hsa-miR-3173-5p

hsa-miR-1276

hsa-miR-193b-3p

hsa-miR-125b-5p

hsa-miR-30c-5p

hsa-miR-296-3p

hsa-miR-30d-5p

hsa-miR-193a-3p

hsa-miR-654-5p

hsa-miR-125a-5p

hsa-miR-1301-3p

hsa-miR-1295a

hsa-miR-139-5p

hsa-miR-495-3p

hsa-miR-541-3p

hsa-miR-510-5p

hsa-miR-206

hsa-miR-654-3p

hsa-miR-130a-5p

hsa-miR-4731-5p

hsa-miR-501-3p

hsa-miR-92a-3p

hsa-miR-1343-3p

hsa-miR-421

hsa-miR-148b-3p

hsa-miR-3180-3p

hsa-miR-367-3p

hsa-miR-2278

hsa-miR-3187-3p

hsa-miR-32-5p

hsa-miR-148a-3p

hsa-miR-502-3p

hsa-miR-876-5p

hsa-miR-942-5p

hsa-miR-1193

hsa-miR-503-5p

hsa-miR-483-3p

hsa-miR-152-3p

hsa-miR-25-3p

hsa-miR-363-3p

hsa-miR-92b-3p

hsa-miR-365b-3p

hsa-miR-337-3p

hsa-miR-671-5p

hsa-miR-24-3p

hsa-miR-299-3p

hsa-miR-3622b-5p

hsa-miR-216a-3p

hsa-miR-5691

hsa-miR-370-5p

hsa-miR-33b-5p

hsa-miR-3200-5p

hsa-miR-374a-5p

hsa-miR-452-5p

hsa-miR-197-3p

hsa-miR-411-5p

hsa-miR-96-5p

hsa-miR-124-3p

hsa-miR-22-3p

hsa-miR-506-3p

hsa-miR-1271-5p

hsa-miR-182-5p

hsa-miR-656-3p

hsa-miR-4524a-5p

hsa-miR-455-3p

hsa-miR-1913

hsa-miR-324-5p

hsa-miR-145-5p

hsa-miR-519e-5p

hsa-miR-376a-3p

hsa-miR-28-5p

hsa-miR-149-5p

hsa-miR-184

hsa-miR-185-5p

hsa-miR-212-5p

hsa-miR-129-2-3p

hsa-miR-335-5p

hsa-miR-483-5p

hsa-miR-138-5p

hsa-miR-3064-5p

hsa-miR-140-3p

hsa-miR-181a-5p

hsa-miR-496

hsa-miR-650

hsa-miR-7-5p

hsa-miR-545-3p

hsa-miR-376b-3p

hsa-miR-642a-5p

hsa-miR-1323

hsa-miR-155-5p

hsa-miR-515-5p

hsa-miR-708-5p

hsa-miR-208a-3p

hsa-miR-330-3p

hsa-miR-423-3p

hsa-miR-181c-5p

hsa-miR-324-3p

hsa-miR-181d-5p

hsa-miR-3612

hsa-miR-181b-5p

hsa-miR-3121-3p

hsa-miR-1270

hsa-miR-208b-3p

hsa-miR-320b

hsa-miR-3164

hsa-miR-514b-5p

hsa-miR-4766-5p

hsa-miR-516b-5p

hsa-miR-216b-5p

hsa-miR-582-5p

hsa-miR-339-5p

hsa-miR-1179

hsa-miR-524-5p

hsa-miR-223-3p

hsa-miR-377-3p

hsa-miR-331-3p

hsa-miR-200a-3p

hsa-miR-141-3p

hsa-miR-99a-5p

hsa-miR-532-5p

hsa-miR-615-3p

hsa-miR-99b-5p

hsa-miR-100-5p

hsa-miR-382-5p

hsa-miR-296-5p

hsa-miR-29a-3p

hsa-miR-29b-3p

hsa-miR-29c-3p

hsa-miR-143-3p

hsa-miR-214-5p

hsa-miR-383-5p

hsa-miR-625-5p

hsa-miR-486-5p

hsa-miR-491-5p

hsa-miR-766-5p

hsa-miR-522-3p

hsa-miR-802

hsa-miR-455-5p

hsa-miR-2681-3p

hsa-miR-376c-3p

hsa-miR-330-5p

hsa-miR-371a-5p

hsa-miR-4739

hsa-miR-3940-3p

hsa-miR-545-5p

hsa-miR-3126-5p

hsa-miR-4428

hsa-miR-6835-3p

hsa-miR-1306-5p

hsa-miR-3150a-3p

hsa-miR-448

hsa-miR-885-5p

hsa-miR-9-3p

hsa-miR-889-3p

hsa-miR-410-3p

hsa-miR-873-3p

hsa-miR-2467-3p

hsa-miR-224-3p

hsa-miR-6509-3p

hsa-miR-509-3p

hsa-miR-224-5p

hsa-miR-375

hsa-miR-216a-5p

hsa-miR-3681-5p

hsa-miR-651-5p

hsa-miR-186-5p

hsa-miR-425-5p

hsa-miR-590-5p

hsa-miR-655-3p

hsa-miR-21-5p

hsa-miR-4424

hsa-miR-126-5p

hsa-miR-500b-5p

hsa-miR-2681-5p

hsa-miR-744-5p

hsa-miR-2115-3p

hsa-miR-142-5p

hsa-miR-299-5p

hsa-miR-580-3p

hsa-miR-1287-5p

hsa-miR-3150b-3p

hsa-miR-28-3p

hsa-miR-133b

hsa-miR-769-5p

hsa-miR-3194-3p

hsa-miR-1277-5p

hsa-miR-3194-5p

hsa-miR-3690

hsa-miR-493-5p

hsa-miR-877-5p

hsa-miR-1908-5p

hsa-miR-3944-3p

hsa-miR-1197

hsa-miR-874-3p

hsa-miR-342-3p

hsa-miR-663a

hsa-miR-328-3p

hsa-miR-1296-5p

hsa-miR-361-3p

hsa-miR-505-3p

hsa-miR-4640-5p

hsa-miR-494-3p

hsa-miR-361-5p

hsa-miR-508-3p

hsa-miR-556-5p

hsa-miR-3174

hsa-miR-219a-5p

hsa-miR-378a-3p

hsa-miR-5010-5p

hsa-miR-574-5p

hsa-miR-129-1-3p

hsa-miR-3127-5p

hsa-miR-3614-5p

hsa-miR-6509-5p

hsa-miR-382-3p

hsa-miR-1911-5p

hsa-miR-625-3p

hsa-miR-194-5p

hsa-miR-888-5p

hsa-miR-190a-5p

hsa-miR-624-3p

hsa-miR-641

hsa-miR-144-3p

hsa-miR-101-3p

hsa-miR-652-3p

hsa-miR-151a-5p

hsa-miR-2114-5p

hsa-miR-374c-5p

hsa-miR-500a-3p

hsa-miR-3924

hsa-miR-1269a

hsa-miR-5094

hsa-miR-4766-3p

hsa-miR-1247-5p

hsa-miR-4703-5p

hsa-miR-151b

hsa-miR-202-5p

hsa-miR-4661-5p

hsa-miR-892c-3p

hsa-miR-132-5p

hsa-miR-3942-5p

hsa-miR-3617-5p

hsa-miR-191-5p

hsa-miR-451a

hsa-miR-1307-3p

hsa-miR-1249-3p

hsa-miR-374b-3p

hsa-miR-541-5p

hsa-miR-4761-5p

hsa-miR-374a-3p

hsa-miR-3613-5p

hsa-miR-3611

hsa-miR-624-5p

hsa-miR-323b-3p

hsa-miR-210-3p

hsa-miR-5009-3p

hsa-miR-4640-3p

hsa-miR-499b-5p

hsa-miR-126-3p

hsa-miR-2277-5p
